# GSDMD/Caspase-1-based evaluation of dydrogesterone in preventing recurrence after hysteroscopic endometrial polypectomy

**DOI:** 10.1016/j.clinsp.2026.101026

**Published:** 2026-06-26

**Authors:** Kai Liu, Chunyan Hu, Liang Jia, Yuan Shi, Xinru Wang, Yanni Tian

**Affiliations:** a Department of Gynecology II, Northwest Women's and Children's Hospital, Xian, Shaanxi, China; b Department of Women's Healthcare, Northwest Women's and Children's Hospital, Xian, Shaanxi, China

**Keywords:** GSDMD, Caspase-1, Endometrial polyps, Recurrence, Combined diagnosis

## Abstract

**Objective:**

The research focuses on understanding how GSDMD and Caspase-1, key regulators of pyroptosis, influence postoperative recurrence in Endometrial Polyps (EPs) and examines whether a combined biomarker can effectively predict recurrence likelihood.

**Methods:**

264 EP patients (stratified post-surgery into dydrogesterone-treated and control groups) and 132 healthy women were included. Peripheral blood GSDMD-N, Caspase-1, and estrogen concentrations were quantified. Follow-up assessments at one-year post-operation were conducted to examine associations between these pyroptotic markers and clinical outcomes.

**Results:**

Increases in GSDMD-N and Caspase-1 were observed in EP patients versus healthy subjects (*p* < 0.05). A diagnostic AUC of 0.781 was determined for the joint model. Pyroptosis markers correlated positively with FSH//E_2_ and negatively with P (*p* < 0.05). Recurrent post-surgery cases had higher GSDMD-N/Caspase-1 than non-recurrent ones (*p* < 0.05); the predictive AUC of the combined model for recurrence reached 0.727.

**Conclusion:**

Post-EP recurrence correlates with GSDMD/Caspase-1 levels, and integrating these markers enhances recurrence risk assessment.

## Highlights

• GSDMD-N and Caspase-1 were elevated in EP.
• Dual markers improved EP diagnosis.
• Pyroptosis markers correlated with sex hormones.

## Introduction

Endometrial Polyps (EPs) rank among the most prevalent benign uterine conditions in women. Epidemiological studies indicate a global prevalence ranging from 25% to 35%, with even higher detection rates of 30%–50% among infertile women.^1^^,^^2^ Transcervical Resection of Polyps (TCRP), performed via hysteroscopy, is considered the gold-standard treatment for EPs due to its efficacy in symptom relief and enhancement of reproductive outcomes.^3^ Dydrogesterone, a potent oral progestin, is frequently prescribed postoperatively for EPs given its favorable safety profile, excellent bioavailability, and high tolerability.^4^ Despite this, TCRP procedures are associated with 30%‒50% recurrence rates, often necessitating repeat interventions that compromise patient well-being and strain medical resources.^5^ Identifying reliable post-TCRP recurrence prevention methods and elucidating their mechanisms has thus become a critical clinical priority in gynecology.

Emerging evidence implicates pyroptosis ‒ a Gasdermin (GSDMD)-dependent inflammatory cell death ‒ in endometrial homeostasis dysregulation.^6^ Activated GSDMD, the principal effector of this family, generates transmembrane pores that induce cellular swelling, rupture, and subsequent Interleukin (IL)-1β/IL-18 (pro-inflammatory cytokines) release, amplifying inflammation.^7^ This process is governed by Caspase-1, the pivotal upstream protease activating GSDMD.^8^ Prior studies have identified upregulated GSDMD/Caspase-1 expression in polyp tissues, correlating with aggressive polyp growth.^9^ Yet, whether the two correlate with post-TCRP EP recurrence, as well as how dydrogesterone influences this process, has not been studied.

For the first time, key pyroptosis mediators (GSDMD/Caspase-1) were integrated into the postoperative recurrence assessment framework for EP. A prospective cohort study was conducted to track dynamic alterations in GSDMD/Caspase-1 expression following TCRP surgery, comparing dydrogesterone-treated subjects with untreated controls. By correlating these molecular shifts with clinical recurrence outcomes, the authors explored potential links between dydrogesterone and pyroptosis, offering actionable insights for individualized EP therapeutics. Beyond advancing precision therapy for EPs, this investigation sets a precedent for studying postoperative protocols across benign endometrial pathologies.

## Information and methodology

### Research subjects

A prospective cohort study was conducted in the gynecology department between February 2023 and April 2024. Participants included clinically diagnosed EP patients (EP group) and age-matched healthy controls (control group) from routine health examinations. The institutional review board approved the study protocol, with written informed consent obtained from all participants.

### Criteria for enrollment and exclusion

Study participants were selected based on these parameters:

• Reproductive-aged women (18‒50 years) with documented regular menstrual patterns (21‒35-day intervals) or active pregnancy planning;
• Identified EPs (minimum 5 mm diameter, regardless of number) via transvaginal sonography or hysteroscopy with pathological verification;
• Naive to previous polyp resection procedures, achieving complete excision with basal layer removal and pathology-confirmed negative margin status;
• Absence of recent (3-month) exposure to hormonal therapies (e.g., progestational agents, levonorgestrel-releasing intrauterine system);
• Willingly consented to study participation.

Grounds for exclusion included:

• Concurrent diagnoses of endometrial cancer, atypical hyperplasia, submucosal fibroids, or other cancers;
• Clinically significant hepatorenal insufficiency;
• Uncontrolled acute reproductive tract infections;
• Hematological abnormalities affecting normal clotting function;
• Documented mental health disorders.
• Patients lost to follow-up during the prognosis follow-up.

### Grouping and sample size calculation

Sample size estimation assumed 25% GSDM positivity in controls versus 40% in the EP cohort (RR = 0.625). With α = 0.05 (two-sided), 80% power, and 10% attrition, 132 participants per arm were needed (120 after adjusting for dropouts). Following the selection of EP cases, control group participants were matched based on age and reproductive history.

### Treatment methods

The surgical team, consisting exclusively of experienced attending physicians, performed TCRP on all EP cases. Medication commenced on day-5 post-operation, consisting of dydrogesterone (Abbott, Netherlands; 10 mg tablets) taken bid (1 tablet AM and PM) for 14-day cycles. Three such cycles were completed, spanning the initial three postoperative menstrual periods. A prospective follow-up spanning ≥ 1-year was implemented, requiring participants to attend monthly clinical reviews. The study period concluded in April 2025, with the endpoint defined as EP recurrence ‒ confirmed via hysteroscopy and histopathology ‒ manifesting as Abnormal Uterine Bleeding (AUB) or ultrasound-detected intrauterine lesions.

### Sample collection and testing

Pre-treatment and post-operative (day-5) fasting morning blood samples collected from patients were centrifuged to obtain serum. Serum GSDM-N (the primary active fragment of GSDM protein) and Caspase-1 concentrations were quantified via Enzyme-Linked Immunosorbent Assay (ELISA) using two kits: SEE529Hu02 (Nanjing Saihongrui Biotechnology) for GSDM-N and CSB-E13025h (Wuhan Huamei Biotech) for Caspase-1. Standard solutions were diluted to specified gradients (0, 10, 50, 100, 200 pg/mL) according to the kit manual and dispensed into coated wells. The biotinylated secondary antibody (100 μL per well) was then added, followed by 3,3′,5,5′-Tetramethylbenzidine (TMB) substrate and stop solution. Measurements were taken at 450 nm using a microplate reader. Sample concentrations were derived by plotting OD values (y-axis) against standard concentrations (x-axis). Kit-supplied quality controls (low/medium/high) were included per plate, accepting only batches with < 15% Coefficient of Variation (CV).

Upon admission, venous blood was drawn from EP group subjects to quantify serum Follicle-Stimulating Hormone (FSH), Estradiol (E_2_), and Progesterone (P) via automated chemiluminescent immunoassay. Pre-labeled serum samples underwent machine processing, including reagent dispensing, incubation, washing, and signal measurement. The method utilizes specific antibody-antigen interactions, with acridinium ester tracers producing luminescence intensity directly correlating with hormone concentration. Quality assurance involved daily runs of high/low-concentration controls (CV% under 10%).

### Quality control

All serological and histological analyses were conducted in a centralized laboratory to ensure consistency. Standardized reagents, equipment, and protocols were strictly followed. Dedicated research staff conducted follow-ups using structured questionnaires and electronic medical records to minimize bias. Data entry was verified through dual independent review to guarantee accuracy.

### Statistical methods

Data analysis was conducted in SPSS 30.0. Measurement data distribution was assessed through Shapiro-Wilk normality testing, with normally distributed data presented as (χ±s) and non-normal data as M (P25, P75)]. Between-group comparisons used *t*-tests (normally distributed data) or Mann-Whitney tests (non-parametric data). Categorical variables were expressed as counts (percentages), analyzed using χ² tests or Fisher's exact tests. Receiver Operating Characteristic (ROC) curve analysis evaluated diagnostic performance. Correlation analyses utilized Pearson or Spearman coefficients based on data distribution; p-values below 0.05 indicated significance.

## Results

### Comparability analysis

Statistical comparisons of age, reproductive history, and prior abortions between controls and EP patients demonstrated nonsignificant disparities (*p* > 0.05; SMD < 0.1 for all variables). This indicates balanced group characteristics with clinically irrelevant variations, supporting adequate comparability (Table 1).

**Table 1:** Clinical data sheet for the normal and EP groups.

	Normal group (*n* = 132)	EP group (*n* = 132)	Statistical analysis	Statistics	p
Age	35.30 ± 4.89	35.16 ± 5.08	Independent-samples *t*-test	*t* = 0.234	0.815
History of reproductive			Chi-Square test	χ^2^ = 1.354	0.245
Yes	114 (86.36)	120 (90.91)			
No	18 (13.64)	12 (9.09)			
History of miscarriage			Chi-Square test	χ^2^ = 0.239	0.625
Yes	8 (6.06)	10 (7.58)			
No	124 (93.94)	122 (92.42)			
Body mass index (kg/m^2^)	23.10±1.50	22.81±1.66	Independent-samples *t*-test	t = 1.485	0.139
Marital status			Chi-Square test	χ^2^ = 0.349	0.555
Married	127 (96.21)	125 (94.70)			
Unmarried	5 (3.79)	7 (5.30)			
Place of residence			Chi-Square test	χ^2^ = 0.564	0.453
Cities	75 (56.82)	81 (61.36)			
Rural areas	57 (43.18)	51 (38.64)			
Smoking			Chi-Square test	χ^2^ = 0.510	0.475
Yes	30 (22.73)	35 (26.52)			
No	102 (77.27)	97 (73.48)			

### Comparison of GSDMD-N/Caspase-1 concentrations

**Fig. 1:**
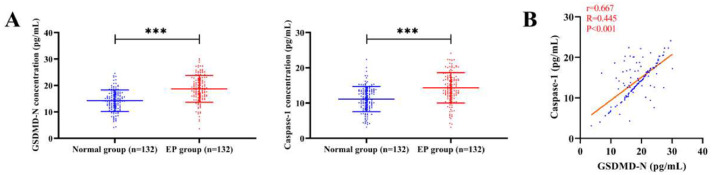
Detection results of GSDMD-N and Caspase-1 in peripheral blood of normal group and EP group (before treatment). (A) Comparison of GSDMD-N and Caspase-1 concentrations in peripheral blood between normal and EP groups. (B) Correlation between GSDMD-N and Caspase-1 concentrations in peripheral blood of EP group (*r* = 0.667). *** *p* < 0.001.

Peripheral blood GSDMD-N and Caspase-1 levels were significantly higher in EP cases than in controls (*p* < 0.05). Besides, statistical analysis using Pearson’s method confirmed a clear positive association between the two biomarkers in EP patients (*p* < 0.05, Fig. 1).

### Clinical significance of GSDMD-N/Caspase-1 in EP diagnosis

**Fig. 2:**
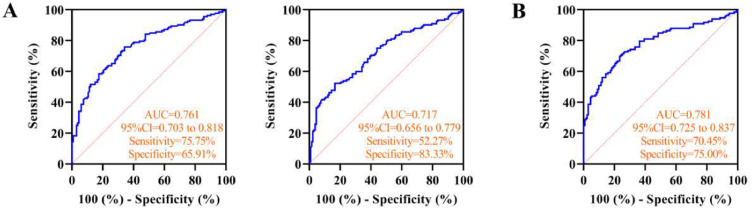
Diagnostic efficacy of GSDMD-N and Caspase-1 for EP (before treatment). (A) ROC curves of GSDMD-N and Caspase-1 for the diagnosis of EP. (B) ROC curve of the combined detection of GSDMD-N and Caspase-1 for the diagnosis of EP.

Based on ROC analysis, GSDMD-N had an AUC value of 0.761 for EP diagnosis, with 75.75% sensitivity and 65.91% specificity (*p* < 0.05). Caspase-1, on the other hand, yielded an AUC of 0.717, along with 52.27% sensitivity and 83.33% specificity (*p* < 0.05). By applying binary logistic regression, a dual-marker model combining GSDMD-N and Caspase-1 was developed. This combined approach reached an AUC of 0.781, showing markedly enhanced diagnostic accuracy compared to individual markers. The model also improved specificity to 70.45% while maintaining 75.00% sensitivity (*p* < 0.05) (Fig. 2).

### Relationship between GSDMD-N/Caspase-1 and estrogen levels in EP patients

**Fig. 3:**
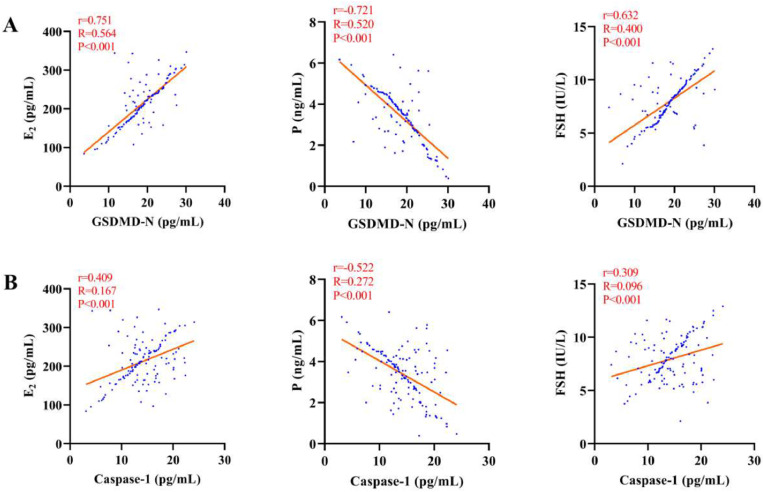
Relationship between GSDMD-N/Caspase-1 and estrogen levels in EP patients. (A) Correlation between GSDMD-N and E_2_/P/FSH. (B) Correlation between Caspase-1 and E_2_/P/FSH.

The EP group demonstrated higher FSH and E_2_ levels but lower P compared to normal subjects (*p* < 0.05). Pearson analysis indicated that GSDMD-N and Caspase-1 expression positively correlated with FSH and E_2_ but inversely correlated with P (*p* < 0.05). In other words, elevated GSDMD-N and Caspase-1 levels correspond to increased FSH and E_2_ but decreased P (Table 2 and Fig. 3).

**Table 2:** Comparison of estrogen levels between the normal and EP groups.

Groups	E_2_ (pg/mL)	P (pg/mL)	FSH (IU/L)
Normal group (*n* = 132)	156.06 ± 39.31	4.92 ± 1.84	7.32 ± 2.06
EP group (*n* = 132)	213.36 ± 56.55	3.38 ± 1.25	7.95 ± 2.04
Statistical analysis	Independent-samples *t*-test		
*T*	9.558	7.947	2.519
P	<0.001	<0.001	0.012

### Preoperative and postoperative variations in GSDMD-N/Caspase-1 levels

Postoperative retesting of GSDMD-N and Caspase-1 levels in the EP group revealed significant reductions in peripheral blood concentrations compared to preoperative measurements (*p* < 0.05, Table 3).

**Table 3:** Comparison of changes in GSDMD-N and Caspase-1 concentrations before and after treatment in the EP group.

Timing	GSDMD-N (pg/mL)	Caspase-1 (pg/mL)
Before treatment (*n* = 132)	18.70 ± 5.07	14.31 ± 4.30
After treatment (*n* = 132)	15.48 ± 2.87	13.33 ± 3.70
Statistical analysis	paired-*t*-test	
*T*	6.340	1.990
P	<0.001	0.048

### Prognostic value of GSDMD-N and CASPASE-1 in predicting EP recurrence

**Fig. 4:**
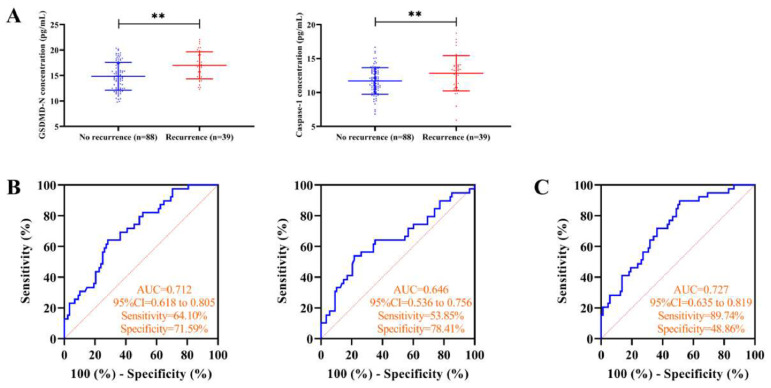
Relationship between GSDMD-N, Caspase-1, and EP prognostic recurrence (after treatment). (A) Comparison of GSDMD-N and Caspase-1 concentrations between patients with prognostic recurrence and patients without recurrence. (B) ROC curve of GSDMD-N and Caspase-1 in predicting the prognosis of EP recurrence. (C) ROC curve of the combined detection of GSDMD-N and Caspase-1 in predicting the prognosis of EP recurrence (**p < 0.01).

The study successfully tracked 127 EP cases, with 39 patients exhibiting recurrence, translating to a 30.7% recurrence rate. The recurrence cases demonstrated elevated GSDMD-N and Caspase-1 concentrations after surgery when measured against non-recurrent cases (*p* < 0.05). Further ROC curve analysis revealed that postoperative GSDMD-N levels predicted EP recurrence with an AUC of 0.712 (sensitivity = 64.10%, specificity = 71.59%, *p* < 0.05), while Caspase-1 exhibited an AUC of 0.646 (53.85% sensitivity, 78.41% specificity; *p* < 0.05). Using postoperative GSDMD-N and Caspase-1 levels, a dual-marker predictive model was similarly developed. For EP recurrence prognosis, it achieved 89.74% sensitivity and 48.86% specificity, with an improved AUC of 0.727 (*p* < 0.05) over single-analyte testing (Fig. 4).

## Discussion

Endometrial Polyps (EPs), a highly prevalent benign uterine lesion in reproductive-aged women, remain a major clinical challenge in gynecological practice due to their high postoperative recurrence rate.^10^ The present study establishes, for the first time, the clinical relevance of the key pyroptosis effectors GSDMD and Caspase-1 in the clinical management of EPs. Cohort analyses identified significantly higher circulating GSDMD-N and Caspase-1 concentrations in EP patients versus controls, with expression patterns influenced by estrogen concentrations. Besides, the dual-marker algorithm proves clinically valuable for both primary diagnosis and recurrence prediction, with the potential to optimize current clinical management strategies for EPs.

GSDMD, the central effector of pyroptosis, generates transmembrane pores upon activation, enabling IL-1β/18 secretion to exacerbate inflammation.^11^ The data reveal upregulated pyroptotic proteins in EPs, consistent with Endometriosis (EMs) studies where ectopic lesions show activated GSDMD/NLRP3 pathways linked to clinical pain and aggressive progression.^12^ Combining these findings with published literature, the authors hypothesize that GSDMD-mediated pyroptosis contributes to EP progression via the following two pathways: 1) Estrogen-mediated activation^13^: High E_2_ concentrations in EP cases stimulate Pro-IL-1β transcription through NF-κB signaling, followed by NLRP3 inflammasome activation and Caspase-1-dependent cleavage of GSDMD-N. 2) P-antagonism.^14^ Through PR-dependent PI3K/Akt signaling, P inhibits Caspase-1 function. However, reduced P in EP cases diminishes this suppression, resulting in prolonged pyroptosis. The study observed that GSDMD-N and Caspase-1 levels correlate positively with FSH and E_2_ but inversely with P, aligning with this mechanism.

For the diagnosis of EPs, GSDMD-N achieved an AUC of 0.761, suggesting robust clinical utility. When combined with Caspase-1, the model's performance further improves (AUC = 0.781), likely due to their synergistic interaction in the pyroptosis pathway.^15^ Notably, patients with recurrence maintained significantly higher pyroptosis-related protein expression than those remaining recurrence-free, implying that prolonged pyroptosis activation could drive disease recurrence. Their combination proved particularly effective for forecasting EP recurrence, introducing novel possibilities for clinical surveillance. This observation mirrors earlier colorectal cancer studies where pyroptosis proteins predicted surgical outcomes.^16^ Peripheral blood-based GSDMD-N/Caspase-1 assays demonstrate higher patient adherence compared to classical EP diagnostic approaches. For prognostic follow-up, repeated blood tests enable dynamic monitoring of pyroptosis protein levels, offering advantages over one-time imaging. Nevertheless, the lack of standardized detection protocols may lead to inter-laboratory variability, and larger cohorts are needed to refine cutoff thresholds.

Prior investigations predominantly examined pyroptosis proteins in oncological contexts. This study, however, demonstrates their abnormal regulation in EPs. Notably, the high-estrogen milieu in EP patients might activate pyroptosis through mechanisms comparable to GSDMD/NF-κB pathway stimulation in PCOS ovarian cells.^17^ Unlike the well-established consensus in oncology that pyroptosis primarily functions as a tumor suppressor^18^, the observations in EPs suggest that pyroptosis may simultaneously drive inflammatory responses and abnormal proliferative activity, underscoring its multifaceted role. Dydrogesterone might regulate pyroptosis through progesterone receptor pathways, but its specific mechanisms in EPs require further investigation via randomized trials. The research findings suggest two clinical translation strategies: 1) Diagnostic enhancement: The combination of GSDMD-N/Caspase-1 biomarkers with established surveillance techniques (ultrasonography and hysteroscopic evaluation) could improve recurrence prediction in EP cases. 2) Individualized medication instruction: Serial assessment of pyroptosis proteins may inform individualized dydrogesterone administration schedules. However, several limitations should be noted. First, despite a total sample size of 264, the recurrence group (*n* = 39) may have been too small for robust subgroup analyses. Second, the 1-year follow-up period was insufficient to capture the long-term dynamic pattern of EP recurrence. Third, without supporting in vitro studies, the authors were unable to examine local endometrial levels of pyroptosis-associated proteins or related inflammatory mediators. The functional involvement of GSDMD/Caspase-1 in EP pathogenesis requires additional exploration. 4) In this study, the authors refer to the hypothesis that E2 enhances NF-κB/NLRP3 and progesterone enhances Caspase-1 via PI3K/Akt is plausible but extrapolated from non-endometrial studies; in vitro experiments using endometrial cells are needed for validation. 5) Although baseline characteristics were comparable between groups, the authors did not perform multivariate modeling to adjust for potential confounding factors that may influence the associations between circulating GSDMD/Caspase-1 levels and EP development, as well as postoperative recurrence. 6) This study only focused on dydrogesterone, without performing head-to-head comparisons with other progestins or anti-inflammatory treatments.

## Conclusion

GSDMD/Caspase-1 are associated with EP recurrence. Combined detection offers non-invasive biomarkers, but larger cohorts and longer follow-up are needed for validation.

## Ethical approval

The study protocol was approved by the Ethics Committee of Northwest Women's and Children's Hospital (Approval number: L-2023–239).

## Consent to publish

All authors gave final approval of the version to be published.

## Authors' contributions

YN.T. designed and supervised the study, K.L. wrote and revised the manuscript, CY.H. and L.J. collected and analyzed data, Y.S. and XR.W. visualized the data, and all authors read and approved the final submitted manuscript.

## Funding

Not applicable.

## Declaration of competing interest

The authors declare no conflicts of interest.

## Data Availability

The data used to support the findings of this study are available from the corresponding author upon request.
